# Polymeric Fibers as Scaffolds for Spinal Cord Injury: A Systematic Review

**DOI:** 10.3389/fbioe.2021.807533

**Published:** 2022-02-09

**Authors:** Yuanpei Cheng, Yanbo Zhang, Han Wu

**Affiliations:** Department of Orthopeadics, China-Japan Union Hospital of Jilin University, Changchun, China

**Keywords:** polymeric fibers, electrospinning technology, neural tissue engineering, spinal cord injury, application

## Abstract

Spinal cord injury (SCI) is a complex neurological condition caused by trauma, inflammation, and other diseases, which often leads to permanent changes in strength and sensory function below the injured site. Changes in the microenvironment and secondary injuries continue to pose challenges for nerve repair and recovery after SCI. Recently, there has been progress in the treatment of SCI with the use of scaffolds for neural tissue engineering. Polymeric fibers fabricated by electrospinning have been increasingly used in SCI therapy owing to their biocompatibility, complex porous structure, high porosity, and large specific surface area. Polymer fibers simulate natural extracellular matrix of the nerve fiber and guide axon growth. Moreover, multiple channels of polymer fiber simulate the bundle of nerves. Polymer fibers with porous structure can be used as carriers loaded with drugs, nerve growth factors and cells. As conductive fibers, polymer fibers have electrical stimulation of nerve function. This paper reviews the fabrication, characterization, and application in SCI therapy of polymeric fibers, as well as potential challenges and future perspectives regarding their application.

## Introduction

Spinal cord injury (SCI) is considered as the main type of central nervous system (CNS) injury, which may lead to paraplegia or quadriplegia and affect the quality of life of patients (Ahuja et al., 2017a; Hall et al., 2021). There are approximately 12,500 new patients of SCI every year in North America based on the National SCI Statistical Center (Hachem et al., 2017). There are two types of SCI: primary injuries that can cause immediate neuronal death and tissue damage, and secondary injuries that often lead to permanent functional impairment. Nerve cells undergo necrosis and apoptosis after SCI (Balentine, 1978; Grossman et al., 2000). The absence of nutritional factors and myelin sheath protein, inflammatory reactions, formation of glial scars, blood flow interruption, and other adverse factors in the lesion results in a microenvironment that inhibits nerve regeneration (Fujita and Yamashita, 2014; Alizadeh et al., 2019). Hence, neural morphological repair and functional recovery after SCI remain challenging for patients and clinicians. Drug therapy and surgery have not yielded satisfying results for SCI. Although drug therapy with methylprednisolone (Fehlings et al., 2017) and nutritional factors (Keefe et al., 2017; Anderson et al., 2018) has shown some benefits, it is difficult to maintain local drug concentrations. Surgery (Wilson et al., 2012) could help to maintain stability and reduce secondary injury. However, the limits of surgical therapy are that it is suitable for patients less than 24 h after SCI, and it cannot change the microenvironment of nerve regeneration (Wilson et al., 2017). New therapies such as cell or nerve transplantation also have their limitations. Cell therapy, including transplantation of olfactory ensheathing cells (Wang et al., 2017; Ursavas et al., 2021), Schwann cells (SCs) (Hosseini et al., 2016), neural stem cells (Xue et al., 2021), and mesenchymal stem cells (MSCs) (Wang et al., 2018), aims to create a favorable microenvironment for nerve regeneration and provide cell replacing sources (Assinck et al., 2017; Ahuja et al., 2017b); however, the rate of survival of the transplanted cells is low (Ahuja and Fehlings, 2016). Nerve autografts or allografts can provide a nerve-specific microenvironment and act as a physical scaffold to bridge the nerve defect (Safa and Buncke, 2016). However, the shortage of donors and graft rejection limit the use of autogenous or allogeneic nerve transplantation in the treatment of SCI (Derby, 2012; Uygun et al., 2012).

**FIGURE 1:**
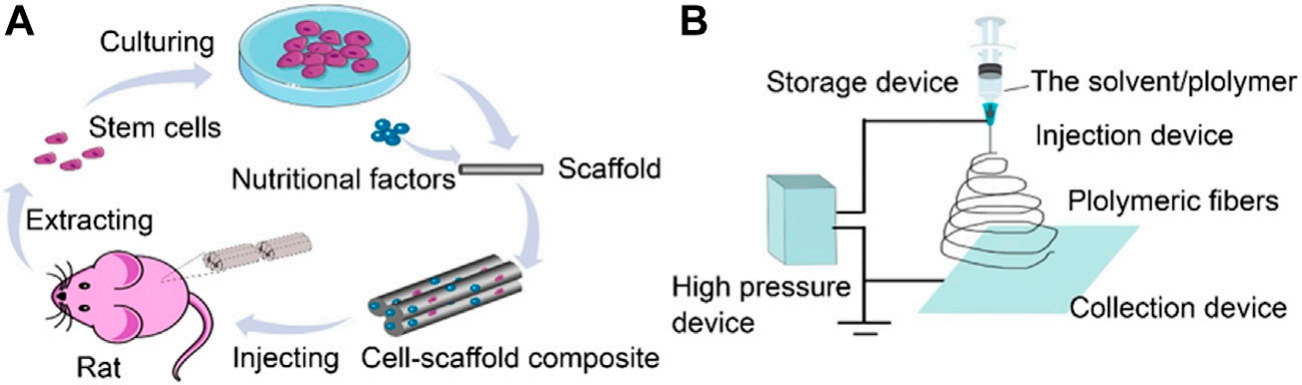
**(A)** Schematic illustration of polymeric fiber scaffolds encapsulating stem cells to be injected into SCI for neural tissue engineering. **(B)** Schematic illustration of electrospinning for polymeric fiber scaffolds.

In recent decades, the application of scaffold-based neural tissue engineering technology has been widely studied for the treatment of SCI (Yang et al., 2004). Seed cells, growth factors, and scaffolds are three major elements of neural tissue engineering. Seed cells commonly used in neural tissue engineering include SCs and stem cells; growth factors promote cell proliferation and differentiation; and scaffolds act as carriers of the seed cells and growth factors for the construction of neural tissues (Figure 1A). Scaffold-based neural tissue engineering aims to fabricate three-dimensional (3D) scaffolds to support and guide the regeneration of nerve tissue in damaged spinal cord defects or spaces (Schmidt and Leach, 2003). The ideal neural tissue scaffold should have good biocompatibility, appropriate biodegradability, excellent mechanical strength, and porosity.

To date, many different types of polymeric fibers have been used as scaffolds for neural tissue engineering. Polymeric fibers can be produced by a variety of processes such as electrospinning (McCullen et al., 2007), phase separation (Liu et al., 2017), self-assembly (Park et al., 2008), melt blowing (Ellison et al., 2007), drawing (Koppes et al., 2016), template synthesis (Liu et al., 2013), and 3D printing (Petcu et al., 2018; Tao et al., 2019) (Table 1). Polymeric fibers prepared by electrospinning are widely used in neural tissue engineering owing to some of its properties such as biocompatibility, biodegradability, high porosity, large specific surface area, high fineness, and homogeneity. Moreover, polymeric fibers have electrical properties that promote nerve regeneration under electrical stimulation (Lee et al., 2009). The structure of electrospun nanofiber scaffolds is similar to that of natural extracellular matrix (ECM), which promotes cell adhesion and proliferation (Tian et al., 2015). Electrospun nanofibers have been shown to promote ganglion growth (Wittmer et al., 2011) and have many applications including drug delivery (Boroojeni et al., 2019) and cell component addition (Baiguera et al., 2014). Nowadays, various natural and synthetic polymers have been prepared by using electrospinning technology for nerve regeneration after SCI. In this review, we summarize the demography and pathophysiology of SCI and present an overview of the fabrication, characterization, and application of polymeric fibers in SCI therapy.

**TABLE 1:** The methods for polymeric fibers fabrication.

Method	Principle	Prous	Cons	Reference
Electrospinning	A method for fabricating nanofiber membranes with diameters ranging from microns to nanometers by accelerating the injection of charged polymer solutions in an electric field	Large selection of materials; adjustable fiber morphology by adjusting electric field strength, flow rate, and spinning head diameter; natural ECM structure and function	The influence of residual solvents; susceptible to interference by ambient temperature, humidityetc.	McCullen et al. (2007)
Phase Separation	It occurs by cooling a homogeneous mixture of polymer and diluent in a hot solution that is solvent-free at room temperature. Phase separation includes dissolution, gelation, extraction using different solvents, freezing and drying to obtain nanofibers	Low cost; high porosity, which facilitates the introduction and release of bioactive components	Time consuming; influence of residual solvents; less controllable morphology	Liu et al. (2017)
Self-Assembly	The precise organization of small and macromolecular building blocks in a non-covalent manner using intermolecular interactions provides a bottom-up approach for the construction of nanofibers	Easy to operate; can mimic natural ECM structure and function; can introduce bioactive factors	Less controllable morphology	Park et al. (2008)
Melt Blowing	Microfibers are produced by injecting a molten polymer stream into a high-speed gas/air jet that forms a self-adhesive web when collected on a moving surface	Simple method; no interference from residual solvents	Vulnerable to ambient temperature, air flow rate	Ellison et al. (2007)
Drawing	Viscoelastic materials that can withstand strong deformation and have sufficient cohesion to support the stresses generated during the drawing process can be made into nanofibers by stretching	Simple process; can be adjusted at any time	Time consuming; uncontrollable morphology; not suitable for all polymers	Koppes et al. (2016)
Template synthesis	Nanofibers are prepared by applying water pressure on one side to pass a polymer solution through pores with nanoscale diameters. Using electrochemical or chemical oxidation polymerization, nanofibers can be produced using nonporous membranes consisting of various cylindrical pores	Controllable diameter of nanofibers	Longer lengths of nanofibers cannot be prepared	Liu et al. (2013)
3D Printing	With additive technologies based on digital design and layer-by-layer precision manufacturing, the entire process no longer requires molds, dies or photolithographic masks, for example. This not only enables a high degree of automation and reproducibility in material manufacturing, but also enables the construction of complex structures	Diversified designs for materials are possible; design structures can be precisely reproduced	Higher cost; high material requirements	Tao et al. (2019)

## Demographics and Pathophysiology

The global incidence of SCI ranges from 3.6 to 195.4 cases per million people (Jazayeri et al., 2015), with 54 per million people being affected in the United States (Jain et al., 2015) and 23.7 (Ning et al., 2011) per million people affected in China. The incidence of SCI has increased in recent years: the incidence of paraplegia and tetraplegia has been reported to be 58.7 and 40.6%, respectively (Rahimi-Movaghar et al., 2013). SCI causes endless pain and heavy economic burden to patients, their families, and the society at large (Ahuja et al., 2017b). As mentioned earlier, SCI are classified as primary and secondary injuries (Kwon et al., 2004). Primary injuries include injuries caused by fractures and fracture dislocation or injuries caused by hyperextension, hyperflexion, and rotation of the spine (Tator, 1995). Acute stage, subacute stage, and chronic stage are the three stages of secondary injury. SCI is immediately followed by the following series of reactions in the acute stage: injury to the blood vessels, ionic imbalance, accumulation of the neurotransmitter (excitotoxicity), formation of free radicals, lipid peroxidation, inflammation, edema, cell necrosis, and cell death (von Leden et al., 2017). The levels of extracellular glutamate increase because of cell death and cytoplasmic content release, leading to glutamate excitotoxicity (Agrawal and Fehlings, 1996). Owing to the excitotoxicity of glutamate, excessive activity of N-methyl-D-aspartic acid receptors results in mitochondrial calcium overload, which could cause cell necrosis (Pivovarova and Andrews, 2010). Subacute injury starts with the development of injury; it includes apoptosis, surviving axonal demyelination, Wallerian degeneration, axonal necrosis, remodeling of the matrix, and the formation of glial scars in the injured area. Ca^2+^ influx activates caspases and some enzymes involved in the decomposition of cytoproteins, resulting in cell apoptosis (Oyinbo, 2011). Further changes that include the formation of cysts, the gradual death of axons, and the maturation of glial scars (Alizadeh and Karimi-Abdolrezaee, 2016; Tran et al., 2018) occur in the chronic stage of injury.

## Preparation and Characterization of Polymeric Fibers

### Preparation of Polymeric Fibers

#### Electrospinning

The formation of polymeric fibers by electrospinning is achieved by overcoming the surface tension of droplets with a high-voltage electrostatic field. A high-pressure device delivers the solvent/polymer located in the solution storage device to the injection device. A polymer droplet forms in which the solvent/polymer remains at the nozzle under the action of electric field and surface tension. With an increasing electric field, the droplets at the nozzle are gradually elongated. When the strength of the electric field increases to a critical value, the force of the electric field will overcome the liquid surface tension and form a transparent conical protrusion called the Taylor cone (Reneker et al., 2000). Subsequently, nanofibers are ejected from the nozzle and scattered randomly in the collection device (Reneker et al., 2000; Reneker et al., 2002). Nevertheless, these nanofibers can be aligned if they are collected between an oscillating collector plate (Fong et al., 2002), a rotating disc (Fennessey and Farris, 2004), or two ground plates (Kakade et al., 2007). Figure 1B shows nanofiber scaffolds prepared by electrospinning.

#### Phase Separation

**FIGURE 2:**
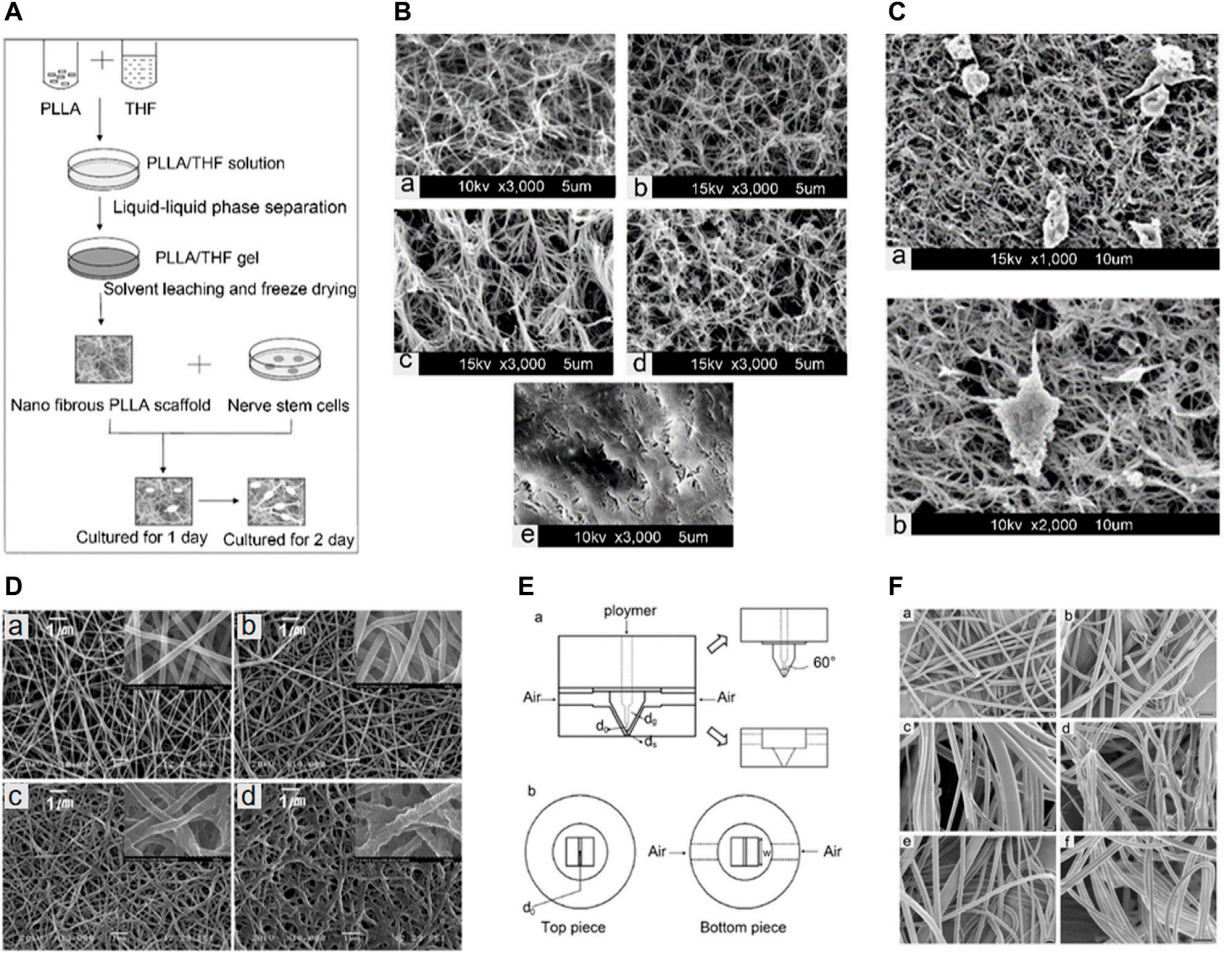
**(A)** Schematic illustration of the fabrication of polymeric fiber scaffolds by phase separation and *in vitro* cell culture. **(B)** SEM micrographs of scaffolds prepared with PLLA/THF concentrations of (a) 2% w/v; (b) 3% w/v; (c) 5% w/v; (d) 7% w/v; and (e) 9% w/v. **(C)** SEM images of PLLA nano-fibrous scaffolds (5% w/v) loaded with C17-2 cells after culture for 1 day: (a) magnification of ×1000; (b) magnified view of a differentiated cell with a short neurite (×2000). Reproduced with permission (Yang et al., 2004). 2003, Elsevier. **(D)** SEM micrographs of polyelectrolyte multilayer-coated nylon 6 fibers with (a) nylon 6 fibers alone (without coating); (b) one bilayer; (c) five bilayers; and (d) 10 bilayers of PSS and PAH. Reproduced with permission (Park et al., 2008). 2007, Wiley. **(E)** Schematic diagram of the melt-blowing die: (a) sectional and (b) end-on views of the two pieces. **(F)** Representative SEM micrographs of the typical fiber mats from (a) PS-1, (b) PS-3, (c) PP-1, (d) PP-3, (e) PBT-1, and (f) PBT-2 melt-blowing runs. Reproduced with permission (Ellison et al., 2007). 2007, Elsevier.

Phase separation is a method that allows the preparation of polymeric fiber scaffolds without any special equipment. There are two types of phase separation: liquid-liquid phase separation and solid-liquid phase separation (Yang et al., 2004). Phase separation typically involves dissolving an appropriate concentration of the polymer in the solvent and preheating the solution until its temperature exceeds its cloud point. The cloud point can be measured by the method described by Matsuyama et al. (Zhang et al., 2020). The solution is quickly placed into a mold and cooled at a low temperature until it becomes a gel. After the gel is freeze-dried, and the solvent is removed, the polymeric fiber scaffold is formed. The preparation of nanostructured scaffolds and the schematic diagram of *in vitro* cell culture are demonstrated in Figure 2A. Polymer concentration has a great influence on the preparation of nanofiber scaffolds. Yang et al. (2004) prepared poly (L-lactic acid) (PLLA) scaffolds by using a liquid-liquid phase separation method. PLLA scaffolds have the structure similar to the natural ECM in the human body. Scanning electron microscopic (SEM) images show that increasing the PLLA concentration increased the average diameter of the fibers, but decreased the porosity and specific surface area of the scaffolds. Figure 2B shows scaffolds prepared with different concentrations of PLLA/tetrahydrofuran (THF). Figure 2C demonstrates that PLLA nanofibrous scaffolds could promoted the differentiation of C17-2 cells. During phase separation, faster cooling rates have been shown to form polymeric fiber scaffolds with smaller pores (Matsuyama et al., 2000).

#### Self-Assembly

Self-assembly is commonly used to prepare nanofibers. The polypeptide nanofiber scaffold prepared by self-assembly is a new biomaterial-based scaffold, which shows promise in nerve repair and regeneration (Ellis-Behnke et al., 2006). In the process of self-assembly, amphiphilic polyelectrolytes (Yan et al., 2008) are prepared first, following which nanofibers are spontaneously formed by physical adsorption. Combining self-assembly with electrospinning can facilitate the preparation of nanofiber scaffolds with better properties. The microstructure of an electrospun nanofiber mat is shown in Figure 2D.

#### Melt Blowing

Melt blowing is commonly used for the preparation of nanofibers, wherein the polymer is placed in the melt-blown mold and heated for conversion into polymer melt. This polymer melt is melt-blown at the mouth of a tubular capillary rheometer, where under the action of hot air in the V-shaped groove, it will gradually decay into fibers that will be scattered in the collection device. The schematic diagram of a single-hole, melt-blown mold is shown in Figure 2E. There are four basic process parameters for melt spraying: the temperature and mass flow rate of the polymer, and the temperature and mass flow rate of air. The mass flow rates of air and polymer are the main factors affecting the size of the fiber. Fibers of different diameters are shown in Figure 2F.

#### Other Technologies

In addition to the above techniques, drawing (Koppes et al., 2016), template synthesis (Liu et al., 2013), and 3D printing (Petcu et al., 2018; Tao et al., 2019) are also widely used in the preparation of polymeric fibers.

### Characterization of Polymeric Fibers

The ideal polymer fiber scaffold has the following characteristics: (Ahuja et al., 2017a) biocompatibility, (Hall et al., 2021), biodegradability, (Hachem et al., 2017), mechanical properties, and (Balentine, 1978) topographical clues.

#### Polymeric Material Selection/Surface Modification

The selection of materials may affect the extension of neurites. Recently, focus has shifted to electrospun polymers, such as poly (glycolic acid) (PGA), polycaprolactone (PCL), poly (lactic acid) (PLA), and poly (lactic acid-co-glycolic acid) (PLGA), for the treatment of SCI. A variety of natural polymers such as collagen and gelatin have also been extensively studied (Schiffman and Schauer, 2008). Fibers with special surface features have been created by modifying the surface chemistry, by combining polymers, or by surface modification to enhance the regeneration potential of nerves. Proteins or peptide sequences can be mixed with biodegradable and synthetic polyesters before electrospinning, so as to integrate the biological molecules into the fibers. For example, PCL aligned nanofibers combined with post-electrospinning surface modification with Gly-Tyr-Ile-Gly-Ser-Arg peptide were useful in stem cell therapy (Silantyeva et al., 2018). Covalent protein coupling or integrin-binding peptide-coupling can apply bioactive molecules to the surface of the fibers to promote neurite elongation (Kim and Park, 2006; Xie et al., 2010).

### Biocompatibility

Biocompatibility is the crucial property of fiber scaffolds in tissue engineering. The implanted fiber scaffolds are foreign substances to the spinal cord, which may cause immune rejection reaction and thus reduce their efficacy (Al-Maawi et al., 2017). Therefore, the fiber scaffolds need to have good biocompatibility to avoid immune rejection. Natural polymer fibers such as gelatin, with high biocompatibility and low immunogenicity (Fan et al., 2018a), are widely used in tissue engineering. A study demonstrated that chitosan had good biocompatibility, did not produce an immune response, and could be used in the treatment of SCI (Kim et al., 2011). PCL/gelatin nanofibers prepared by electrospinning are similar to natural collagen fibers and also have good biocompatibility (Lim et al., 2021). The biocompatibility of polymer fibers can be modulated by surface modification. A recent study concluded that the biocompatibility of PCL/chitosan scaffolds was improved by surface modification (Habibizadeh et al., 2021).

### Polymeric Fiber Geometry

The function of polymeric fiber scaffold depends on its geometry. Electrospun nanofiber scaffolds have high specific surface area (Lin et al., 2013; An et al., 2017), porous structure (Dayal et al., 2007) and core-shell structure (Xu et al., 2006), and these properties enable the fiber scaffolds to load drugs, and bioactive molecules and cells, rendering the fiber scaffolds suitable for wide use in tissue engineering to treat SCI. Nanofiber scaffolds resemble ECM in their structure, have very high specific surface area, and can interact with cells (Lin et al., 2013) to promote cell adhesion and proliferation. Electrospun nanofiber scaffolds also possess the properties of high fiber fineness, good uniformity, and oriented alignment and topographical clues; therefore, the fiber scaffolds can not only bridge nerve defects, but also guide axon directional regeneration. A study conducted by Yang et al. (2005) showed that the aligned electrospun fibers had better contact guidance for neurite growth compared to those fibers at random. And oriented electrospun fibers were found to promote axonal regeneration after acute SCI in experimental models (Hurtado et al., 2011). Another study demonstrated that aligned fibers promoted neurite growth and faster migration of astrocytes (Zuidema et al., 2014). Oriented microtubules had similar physiological structure as the spinal cord and were able to guide axonal regeneration (Huang et al., 2017).

### Drug Delivery

Electrospinning fibers can be used to deliver therapeutic drugs. Local and sustained release of therapeutic drugs integrated into biomaterial scaffolds has been used to promote nerve regeneration or reduce secondary injury after SCI. Drugs are typically implanted into these scaffolds by embedding the polymer matrix in the manufacturing process or by attaching molecules to the surface of the fiber. Drugs are typically implanted into these scaffolds in two ways: one is to embed the polymer matrix during the manufacturing process, the other is to attach molecules to the surface of the fiber (Meinel et al., 2012). The simplest way to add therapeutic molecules to electrospun fibers is to add therapeutic molecules directly to the solution before electrospinning, so as to guarantee a sustained release curve of the drugs (Duque Sánchez et al., 2016). Therapeutic molecules, including growth factors and other proteins, have been used to promote axonal regeneration, plasticity, degradation or removal of inhibitors, and neuroprotection after SCI. High specific surface area and porous structure can carry drugs efficiently (Li et al., 2015). Core shell fibers prepared by coaxial electrospinning and emulsion electrospinning can maintain protein activity (Duque Sánchez et al., 2016), with such nanofibers providing a typical biphasic drug-release curve, including immediate release and sustained release (Hu et al., 2016). In 2005, Chew et al. (2005) first added therapeutic agents during electrospinning for nerve regeneration. Subsequently, Valmikinathan et al. (2009) added bovine serum albumin to an electrospinning solution including nerve growth factor (NGF) and PCL. Yang et al. (2015a) demonstrated that neurotrophin-3 can effectively promote neuronal differentiation of endogenous neural stem cells (NSCs), eventually leading to the recovery of sensory and motor behavior in a completely transected rat SCI model. A recent study demonstrated controlled release of dexamethasone sodium phosphate from a PCL/gelatin scaffold, thus promoting axonal growth, avoiding the formation of a glial scar, inhibiting the proliferation of astrocytes, and reducing the apoptosis of oligodendrocytes for SCI repair (Boroojeni et al., 2019).

## Polymeric Fibers for SCI

Many polymeric fibers have been used in SCI, including natural polymeric fibers, synthetic polymeric fibers, and polymers containing mixtures of natural and synthetic components (Table 2).

**TABLE 2:** Natural and synthetic polymeric fibers for SCI repair.

Material	Animal	Injury type	Outcome	Reference
Collagen	Rat	Transection	Promoting axon regeneration and neurological recovery after SCI	Sun et al. (2019)
	Rat	Hemisection	Decreasing of glial scarring and collagen deposition, and increasing of neurons	Breen et al. (2017)
	Mice	Transection	Connection of stumps in the transected spinal cord, differentiation of transplanted cells	Sugai et al. (2015)
Gelatin	Rat	Transection	Reduction of cavity area, collagen deposition and inflammation	Lan et al. (2017b)
	Mice	Hemisection	Reduction in necrosis, Infiltration of leukocytes, and apoptotic cells	Fan et al. (2018b)
Chitosan	Rat	Hemisection	Promoting recovery of locomotor capacity and nerve transduction of the experimental rats	Wu et al. (2018)
PLA	Rat	Transection	Reducing the activation of astrocytes and increased axonal regeneration	Shu et al. (2019)
	Rat	Transection	Robusting regeneration of vascularized central nervous system tissue	Hurtado et al. (2011)
	Rat	Hemisection	Supporting cell migration, proliferation and axonal regeneration	Cai et al. (2007)
	Rat	Transection	Promoting axonal growth and enhanced the functional recovery	Hurtado et al. (2006)
PCL	Rat	Transection	Promoting axon regeneration in rat SCIs	Shahriari et al. (2017)
	Rat	Hemisection	Restoring the continuity of the injured spinal cord and decreasing cavity formation	Terraf et al. (2017)
	Rat	Compression	Local application of MDL28170-loaded PCL film improved functional recovery by preserving survival of motor neurons after traumatic SCI	Shi et al. (2019)
	Rat	Transection	Promoting axonal growth and enhanced the functional recovery following SCI	Zhang et al. (2018)
PLGA	Rat	Transection	Inducing short-term nerve regeneration and functional recovery following sciatic nerve transection in rats	dos Santos et al. (2019)
	Rat	Hemisection	Promoting angiogenesis and neural regeneration in the injured area	Wen et al. (2016)
	Mice	Hemisection	Improving tissue regeneration, angiogenesis, and the recovery of locomotor function at the injury site	Gwak et al. (2016)

### Natural Polymeric Fibers

Natural polymeric fibers, such as collagen, gelatin, elastin, chitosan and fibroin, have been used to prepare scaffolds. Owing to their biocompatibility, biodegradability, and beneficial effects on cell adhesion and survival, natural polymeric fibers have been widely used to develop various forms of regenerative scaffolds for SCI repair (Libro et al., 2017).

#### Collagen

**FIGURE 3:**
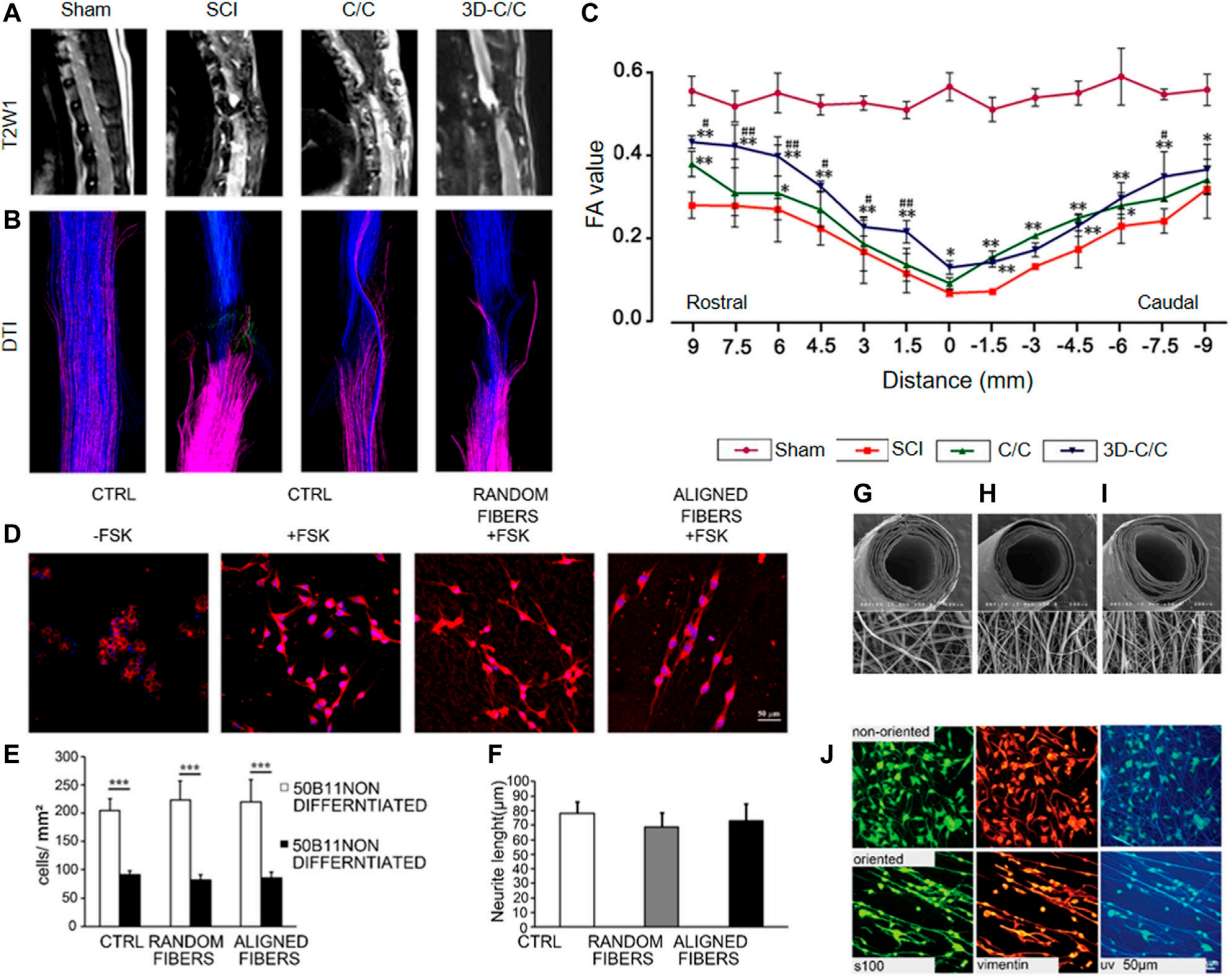
MRI-DTI estimating the recovery of white matter fiber after SCI. **(A)**: T2W1 micrograph of the conventional sagittal spinal cord. **(B)**: DTI-tracked white matter fibers of spinal. **(C)**: The relationship of fractional anisotropy (FA) value and distance (mm). C/C: collagen/chitosan scaffold with freeze drying technology. 3D-C/C: collagen/chitosan scaffold with 3D printing technology. *p < 0.05, **p < 0.01 versus SCI group. ^#^ *p* < 0.05, ^##^ *p* < 0.01 versus C/C group. Reproduced with permission (Sun et al., 2019). 2019, Wiley. **(D)** Confocal micrographs of B5011 cells stained by DAPI (blue) and β-tubulin (red) 24 h after different treatments. **(E)** Comparative 50B11 cell number of different treatment groups. **(F)** Comparative Neurite length of different treatment groups. FSK: forskolin; ***p ≤ 0.001; Scale bar: 50 μm. Reproduced with permission (Gnavi et al., 2015). Copyright 2015, MDPI. **(G)** SEM images of electrospun, non-oriented chitosan nanofiber mesh tube. **(H)** SEM images of electrospun, oriented chitosan nanofiber mesh tube. **(I)** SEM images of electrospun, bilayered chitosan nanofiber mesh tube. **(J)** Immunocytochemistry of the immortalized Schwann cell line, IMS32, after culture for 4 days on the nonoriented (upper) and oriented (lower) chitosan nanofiber mesh sheet. Reproduced with permission (Wang et al., 2009). 2008, Wiley.

Collagen is a ubiquitous protein in the human body and because of its biocompatibility and biodegradability, it is a suitable natural polymer scaffold material for SCI repair (Dong and Lv, 2016). Natural polymeric scaffolds have cell adhesion sites and can be covalently modified (Wissink et al., 2001; Mahoney et al., 2006). The fiber structure of collagen is conducive for cell adhesion, growth, and reproduction (Li and Dai, 2018). Sun et al. (2019) successfully prepared a new collagen-chitosan scaffold by 3D printing technology. This scaffold could partially reconstruct the microenvironment of axonal regeneration and not only reduce the formation of scars and voids, but also promote the regeneration and functional recovery of nerve fibers in rats (Figures 3A–C). In addition, Li et al. proved that implantation of paclitaxel-modified collagen scaffolds could promote endogenous neurogenesis, electrophysiology, and motor recovery in a canine model of acute T8 whole spinal cord transection (Yin et al., 2018). Recent studies have shown that collagen-based paclitaxel or human bone marrow stromal cells (hBMSCs) can further promote locomotor function. Dogs treated with paclitaxel-and hBMSCs-modified nerve regeneration scaffolds showed higher levels of endogenous neuron regeneration in the lesion area (Liu et al., 2019).

#### Gelatin

Gelatin is a natural polymer whose scaffold has good porosity, showing *in vitro* sustained release when modified with a neurotrophin-3 (NT-3)/fibroin complex (Li et al., 2018a). Gelatin scaffolds with porous structure can load basic fibroblast growth factor (bFGF) and continuously release drugs (Lan et al., 2017a). *In vitro* experiments showed that the photo-crosslinked hydrogel microfibril scaffolds could not only provide a good environment for cell survival and metabolism, but also promoted cell proliferation, migration, and differentiation, as well as directional extension of axons (Chen et al., 2019). NT-3 delivery from bioactive scaffolds significantly inhibited inflammation, produced a favorable environment to improve the regeneration of nerve fibers, caused host tissue cells to migrate to injured/transplanted sites to form myelin sheaths and blood vessels, and ultimately increased amplitude of the paralysis hindlimb movement, and shortened delays that increased motor evoked potential of lower limb motor cortex (Li et al., 2018a). A study by Kriebel et al. (2017) demonstrated that electrospun gelatin scaffolds promoted SC migration and axonal regeneration. Gnavi et al. (2015) indicated that gelatin scaffolds prepared by electrospinning appeared to be aligned nanofibers that could induce SCs and axons to grow in a particular direction. However, the proliferation rate of SCs in these oriented fibers was slower than in random fibers (Figures 3D–F).

#### Chitosan

Chitosan is a biological material with good biocompatibility and biodegradation that can promote nerve regeneration (Wu et al., 2018). Electrospun chitosan nanofibers with fiber orientation resulted in SCs lining up along the nanofibers and promoted nerve regeneration (Figures 3G–J) (Wang et al., 2009). Chitosan had a significant effect on neuroprotection and physiological recovery after SCI (Cho et al., 2010). Chitosan conduits loaded with BMSCs promoted the regeneration of nerves and the recovery of nerve function (Chen et al., 2011). Sun et al. (2017a) showed that the chitosan/NT-3 scaffolds with porous surface structure and core-shell structure had little influence on the proliferation of human umbilical cord MSCs (hUC-MSCs). These researchers showed that the chitosan scaffolds infused with NT-3 and hUC-MSCs could inhibit the activation of microglia and reduce the inflammatory reaction after SCI. Yang et al. (2015b) demonstrated that NT3-chitosan induced the activation of endogenous NSCs in the injured spinal cord and promoted the recovery of sensory and motor behaviors after SCI. A recent study demonstrated that the NT3-chitosan scaffolds not only prevented the infiltration of inflammatory cells, but also promoted the differentiation of endogenous NSCs into neurons (Rao et al., 2018).

### Synthetic Polymeric Fibers

Although natural polymeric fibers have demonstrated some beneficial effects as scaffolds in SCI, they possess limitations such as poor mechanical properties, rapid degradation, and poor adjustability. To compensate for the defects of natural polymeric fibers, synthetic polymeric fibers have been prepared and widely used because of their advantages such as good mechanical properties and adjustability.

#### PCL

PCL scaffolds are made up of biocompatible and biodegradable aliphatic polyester and have been widely used in many biomedical applications including the delivery of bioactive drugs for spinal cord regeneration (Wang et al., 2015). PCL promotes the differentiation and myelination of oligodendrocytes in axons and is therefore a suitable material for SCI repair (Donoghue et al., 2013; Patel et al., 2019). Moreover, aligned poly (ε-caprolactam) nanofibers have been found to guide the orientation and migration of neurons, astrocytes, and oligodendrocyte precursors that were derived from human pluripotent stem cells *in vitro* (Hyysalo et al., 2017). Aligned Gly-Tyr-Ile-Gly-Ser-Arg peptide-functionalized nanofibers could accelerate neuronal differentiation of mouse embryonic stem cells (Silantyeva et al., 2018). PCL scaffolds loaded with NT-3 and NSCs could not only stimulated the repair of injured spinal cord nerves, but also promoted the recovery of motor function (Hwang et al., 2011). PCL scaffolds with bioactive factors could guide the regeneration of nerve fibers along the pores of scaffolds, promote angiogenesis, and restore movement and function (Gelain et al., 2011). A study revealed that astragoloside IV-PCL (AST-PCL) could markedly inhibit apoptosis and reduce tissue damage as well as promote functional recovery of rats with SCI (Zhang et al., 2019). In addition, PCL induced neural differentiation of stem cells through different topological structures, which is very important for SCI repair. Mohtaram et al. (2015) demonstrated the positive effects of different topological structures of electrospun retinoic acid-PCL (RA/PCL) scaffolds on human induced pluripotent stem cells (hiPSCs) in neuronal differentiation.

#### PLA

**FIGURE 4:**
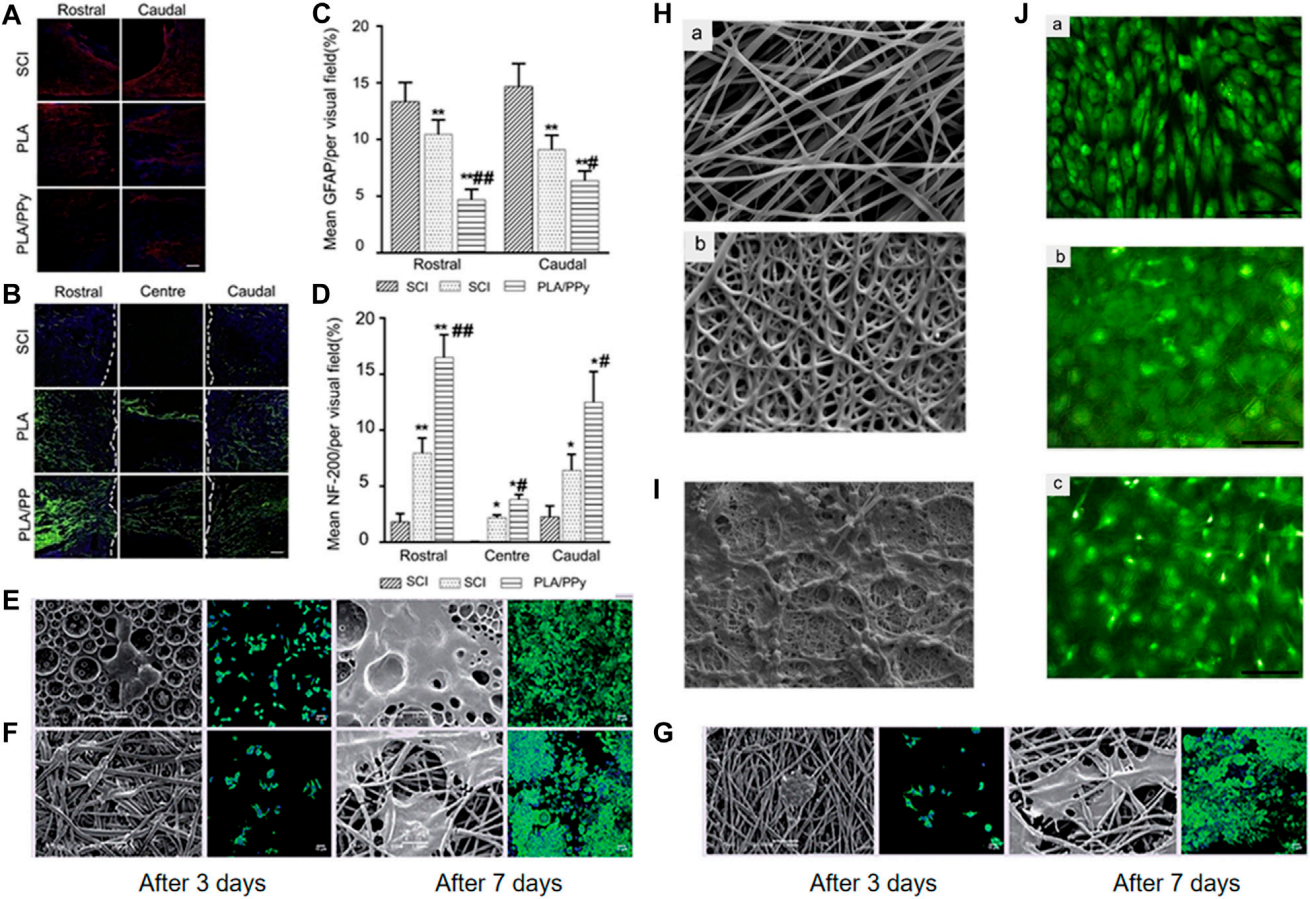
Astrocyte activation decreased and axonal regeneration increased after 6 weeks of injury. Immunostaining images of surrounding tissues stained by **(A)** antiglial fibrillary acidic protein (GFAP) (red) and **(B)** NF200 (green) antibody in different groups 6 weeks after injury. Quantitative analysis of axons stained by **(C)** GFAP and **(D)** NF200-positive in different groups. *p < 0.05, **p < 0.01. *p < 0.05 versus SCI group, **p < 0.01 versus SCI group. #*p* < 0.05 versus PLA group, ^##^ *p* < 0.01 versus PLA group. Scale bar = 100 μm. Reproduced with permission (Shu et al., 2019). 2019, Elsevier. Scanning electron microscopy and fluorescence microscopy micrographs of PC12 cells cultivated on different scaffolds after culture for 3 and 7 days: **(E)** PLGA film; **(F)** PLGA scaffold; **(G)** PLGA/FGF-2 scaffold. Reproduced with permission (Reis et al., 2018). 2018, Future Science. **(H)** SEM micrographs of electrospun (a) PCL and (b) PCL/gelatin scaffolds. **(I)**. SEM micrographs of the morphology of hiPSCs loaded on PCL/gelatin scaffolds after 14 days. **(J)** Fluorescence microscopy micrographs of cells stained by acridine orange and grown in (a) control sample, (b) PCL nanofibers, and (c) PCL/PEG nanofiber scaffolds for 3 days. Reproduced with permission (KarbalaeiMahdi et al., 2017). 2017, Elsevier.

PLA is a biocompatible lactic acid polymer (Gautier et al., 1998). Several PLA preparations have been approved by the United States-Food and Drug Administration for the treatment of SCI. Transplants containing neatly arranged PLA microfibers promoted the regeneration of CNS tissues, consisting of axons and glial cells, from neurons in the spinal cord and propriospinal cord (Hurtado et al., 2011). Polydopamine-PLGA/NGF scaffolds not only promoted the proliferation and neuronal differentiation of NSCs *in vitro*, but also promoted the recovery of SCI *in vivo* (Pan et al., 2019a). Conductive polypyrrole (PPy) was embedded in electrospun PLA nanofiber scaffolds (PLA/PPy scaffolds) to prepare a composite biomaterial. The scaffold was implanted into the spinal cord of rat T9 and completely resected. It is noteworthy that the PLA/PPy scaffolds significantly reduced astrocyte activation and increased the regeneration of axons after 6 weeks of injury (Figures 4A–D) (Shu et al., 2019).

#### PLGA

PLGA copolymer is the product of the reaction between PGA and PLA, both biodegradable, synthetic polymers (Nomura et al., 2006). PLGA scaffolds have good porosity, hydrophilicity, and biodegradability and can also be used as drug carriers. The drug delivery device is a PLGA-based nerve conduit, which is used to control the local delivery of NGF, and is applied to the peripheral nerve gap injury (Labroo et al., 2017). A study of an animal model of SCI showed that the local delivery of insulin-like growth factor 1 and brain-derived neurotrophic factor immobilized on graphene oxide (GO)-incorporated PLGA (PLGA/GO) nanofibers markedly improved functional recovery and increased the number of neurons in the injured sites (Pan et al., 2019b). The uniform microfibers produced by electrospinning had core-shell structure, and fibroblast growth factor-2 (FGF-2) in the fibers was released in a sustained manner. The fibers could support the adhesion and proliferation of pheochromocyte 12 (PC12) cells. On the 28th day after SCI, scaffold implantation was found to promote motor recovery and reduce the expression of antiglial fibrillary acidic protein (GFAP) (Figures 4E–G) (Reis et al., 2018). PLGA scaffolds have been found to promote SC differentiation and spinal cord recovery (Sun et al., 2017b). PLGA/polyethylene glycol (PEG) nanofibers induce pluripotent stem cells to produce neural precursor cells for SCI repair (Pang et al., 2016).

### Polymers Consisting of Natural and Synthetic Components

Polymer fiber composites composed of several kinds of polymers can compensate for the shortcomings of a single material. PCL/chitosan scaffolds formed by electrospinning show biocompatibility and low cytotoxicity (Bolaina-Lorenzo et al., 2016). Electrospun PCL/collagen/elastin nanofibers had good physical, chemical, and mechanical properties (Aguirre-Chagala et al., 2017). A laminin-chitosan PLGA neural conduit showed good adhesive property and was conducive to nerve regeneration (Li et al., 2018b). PCL/gelatin nanofibers induced hiPSCs to differentiate into neurons (Figures 4H–J) (KarbalaeiMahdi et al., 2017), and PCL-collagen VI could promote the regeneration and functional recovery of nerves (Lv et al., 2017).

## Conclusion

In this review, we summarize the utility of polymeric fibers as scaffolds for the treatment of SCI. Due to its good biocompatibility, biodegradability, high specific surface area, and high porosity and topographic clues, polymeric fibers prepared by electrospinning have been used as scaffolds in neural tissue engineering. Electrospun scaffolds not only provide support and guidance for axonal regeneration, but also enable local release of bioactive molecules to regulate cellular activity and inflammation response, promote angiogenesis, and inhibit the formation of glial scars. Natural polymeric fibers are widely used in neural tissue engineering due to good biocompatibility and biodegradability. However, the low mechanical strength of natural polymeric materials as scaffolds limits its development. Synthetic polymeric fibers are gradually used in the treatment of SCI due to their good mechanical properties. Nevertheless, synthetic polymer fibers also have some limitations, such as poor biocompatibility, poor cell adhesion and low cell affinity. To improve biocompatibility, synthetic polymeric fibers can be combined with natural polymeric fibers by chemical cross-linking or surface modification. The composite polymer fiber scaffolds have good physical, chemical and biological properties.

Currently, in addition to polymeric fiber scaffolds, stem cells and bioactive molecules play an important role in neural tissue engineering. Combination therapy with polymeric fiber scaffolds, stem cells and bioactive molecules is a promising direction for the treatment of SCI. Neural tissue engineering of SCI needs to be further studied in-depth, primarily including the following aspects: 1) the biocompatibility of polymeric fiber scaffolds, stem cells and nerve tissues *in vivo*; 2) the mechanism of differentiation of stem cells into different cell phenotypes; 3) the degradative speed of polymeric fiber scaffolds were kept up with nervous tissue regeneration; and 4) local release curve of drugs loaded on polymeric fiber scaffolds.

In conclusion, polymeric fibers have great potential as scaffolds in neural tissue engineering for the treatment of SCI. We believe that the application of polymeric fiber scaffolds in the treatment of SCI will eventually achieve good clinical results.

## References

[B1] Agrawal S. Fehlings M. (1996). Mechanisms of Secondary Injury to Spinal Cord Axons *In Vitro*: Role of Na+, Na(+)-K(+)-ATPase, the Na(+)-H+ Exchanger, and the Na(+)-Ca2+ Exchanger. J. Neurosci. 16 (2), 545–552. 10.1523/jneurosci.16-02-00545.1996 8551338 PMC6578655

[B2] Aguirre-Chagala Y. E. Altuzar V. León-Sarabia E. Tinoco-Magaña J. C. Yañez-Limón J. M. Mendoza-Barrera C. (2017). Physicochemical Properties of Polycaprolactone/collagen/elastin Nanofibers Fabricated by Electrospinning. Mater. Sci. Eng. C 76, 897–907. 10.1016/j.msec.2017.03.118 28482605

[B3] Ahuja C. S. Fehlings M. (2016). Concise Review: Bridging the Gap: Novel Neuroregenerative and Neuroprotective Strategies in Spinal Cord Injury. Stem Cell Transl Med 5 (7), 914–924. 10.5966/sctm.2015-0381 PMC4922857 27130222

[B4] Ahuja C. S. Nori S. Tetreault L. Wilson J. Kwon B. Harrop J. (2017). Traumatic Spinal Cord Injury-Repair and Regeneration. Neurosurgery 80 (3S), S9–S22. 10.1093/neuros/nyw080 28350947

[B5] Ahuja C. S. Wilson J. R. Nori S. Kotter M. R. N. Druschel C. Curt A. (2017). Traumatic Spinal Cord Injury. Nat. Rev. Dis. Primers 3, 17018. 10.1038/nrdp.2017.18 28447605

[B6] Al-Maawi S. Orlowska A. Sader R. James Kirkpatrick C. Ghanaati S. (2017). *In Vivo* cellular Reactions to Different Biomaterials-Physiological and Pathological Aspects and Their Consequences. Semin. Immunol. 29, 49–61. 10.1016/j.smim.2017.06.001 28647227

[B7] Alizadeh A. Dyck S. M. Karimi-Abdolrezaee S. (2019). Traumatic Spinal Cord Injury: An Overview of Pathophysiology, Models and Acute Injury Mechanisms. Front. Neurol. 10, 282. 10.3389/fneur.2019.00282 30967837 PMC6439316

[B8] Alizadeh A. Karimi-Abdolrezaee S. (2016). Microenvironmental Regulation of Oligodendrocyte Replacement and Remyelination in Spinal Cord Injury. J. Physiol. 594 (13), 3539–3552. 10.1113/JP270895 26857216 PMC4929323

[B9] An A. K. Guo J. Lee E.-J. Jeong S. Zhao Y. Wang Z. (2017). PDMS/PVDF Hybrid Electrospun Membrane with Superhydrophobic Property and Drop Impact Dynamics for Dyeing Wastewater Treatment Using Membrane Distillation. J. Membr. Sci. 525, 57–67. 10.1016/j.memsci.2016.10.028

[B10] Anderson M. A. O’Shea T. M. Burda J. E. Ao Y. Barlatey S. L. Bernstein A. M. (2018). Required Growth Facilitators Propel Axon Regeneration across Complete Spinal Cord Injury. Nature 561 (7723), 396–400. 10.1038/s41586-018-0467-6 30158698 PMC6151128

[B11] Assinck P. Duncan G. J. Hilton B. J. Plemel J. R. Tetzlaff W. (2017). Cell Transplantation Therapy for Spinal Cord Injury. Nat. Neurosci. 20 (5), 637–647. 10.1038/nn.4541 28440805

[B12] Baiguera S. Del Gaudio C. Lucatelli E. Kuevda E. Boieri M. Mazzanti B. (2014). Electrospun Gelatin Scaffolds Incorporating Rat Decellularized Brain Extracellular Matrix for Neural Tissue Engineering. Biomaterials 35 (4), 1205–1214. 10.1016/j.biomaterials.2013.10.060 24215734

[B13] Balentine J. D. (1978). Pathology of Experimental Spinal Cord Trauma. II. Ultrastructure of Axons and Myelin. Lab. Invest. 39 (3), 254–266. 713490

[B14] Bolaina-Lorenzo E. Martínez-Ramos C. Monleón-Pradas M. Herrera-Kao W. Cauich-Rodríguez J. V. Cervantes-Uc J. M. (2016). Electrospun Polycaprolactone/chitosan Scaffolds for Nerve Tissue Engineering: Physicochemical Characterization and Schwann Cell Biocompatibility. Biomed. Mater. 12 (1), 015008. 10.1088/1748-605X/12/1/015008 27934786

[B15] Boroojeni F. R. Mashayekhan S. Abbaszadeh H. A. (2019). The Controlled Release of Dexamethasone Sodium Phosphate from Bioactive Electrospun PCL/Gelatin Nanofiber Scaffold. Iranian J. Pharm. Res. 18 (1), 111–124. PMC6487400 31089349

[B16] Breen B. A. Kraskiewicz H. Ronan R. Kshiragar A. Patar A. Sargeant T. (2017). Therapeutic Effect of Neurotrophin-3 Treatment in an Injectable Collagen Scaffold Following Rat Spinal Cord Hemisection Injury. ACS Biomater. Sci. Eng. 3 (7), 1287–1295. 10.1021/acsbiomaterials.6b00167 33440517

[B17] Cai J. Ziemba K. S. Smith G. M. Jin Y. (2007). Evaluation of Cellular Organization and Axonal Regeneration through Linear PLA Foam Implants in Acute and Chronic Spinal Cord Injury. J. Biomed. Mater. Res. 83A (2), 512–520. 10.1002/jbm.a.31296 17503492

[B18] Chen C. Tang J. Gu Y. Liu L. Liu X. Deng L. (2019). Bioinspired Hydrogel Electrospun Fibers for Spinal Cord Regeneration. Adv. Funct. Mater. 29 (4), 1806899. 10.1002/adfm.201806899

[B19] Chen X. Yang Y. Yao J. Lin W. Li Y. Chen Y. (2011). Bone Marrow Stromal Cells-Loaded Chitosan Conduits Promote Repair of Complete Transection Injury in Rat Spinal Cord. J. Mater. Sci. Mater. Med. 22 (10), 2347–2356. 10.1007/s10856-011-4401-9 21792742

[B20] Chew S. Y. Wen J. Yim E. K. F. Leong K. W. (2005). Sustained Release of Proteins from Electrospun Biodegradable Fibers. Biomacromolecules 6 (4), 2017–2024. 10.1021/bm0501149 16004440

[B21] Cho Y. Shi R. Borgens R. B. (2010). Chitosan Produces Potent Neuroprotection and Physiological Recovery Following Traumatic Spinal Cord Injury. J. Exp. Biol. 213 (9), 1513–1520. 10.1242/jeb.035162 20400636

[B22] Dayal P. Liu J. Kumar S. Kyu T. (2007). Experimental and Theoretical Investigations of Porous Structure Formation in Electrospun Fibers. Macromolecules 40 (21), 7689–7694. 10.1021/ma071418l

[B23] Derby B. (2012). Printing and Prototyping of Tissues and Scaffolds. Science 338 (6109), 921–926. 10.1126/science.1226340 23161993

[B24] Dong C. Lv Y. (2016). Application of Collagen Scaffold in Tissue Engineering: Recent Advances and New Perspectives. Polymers 8 (2), 42. 10.3390/polym8020042 PMC6432532 30979136

[B25] Donoghue P. S. Lamond R. Boomkamp S. D. Sun T. Gadegaard N. Riehle M. O. (2013). The Development of a Ɛ-Polycaprolactone Scaffold for Central Nervous System Repair. Tissue Eng. A 19 (3-4), 497–507. 10.1089/ten.tea.2012.0382 22994455

[B26] dos Santos F. P. Peruch T. Katami S. J. V. Martini A. P. R. Crestani T. A. Quintiliano K. (2019). Poly (Lactide-co-glycolide) (PLGA) Scaffold Induces Short-Term Nerve Regeneration and Functional Recovery Following Sciatic Nerve Transection in Rats. Neuroscience 396, 94–107. 10.1016/j.neuroscience.2018.11.007 30452974

[B27] Duque Sánchez L. Brack N. Postma A. Pigram P. J. Meagher L. (2016). Surface Modification of Electrospun Fibres for Biomedical Applications: A Focus on Radical Polymerization Methods. Biomaterials 106, 24–45. 10.1016/j.biomaterials.2016.08.011 27543920

[B28] Ellis-Behnke R. G. Liang Y.-X. You S.-W. Tay D. K. C. Zhang S. So K.-F. (2006). Nano Neuro Knitting: Peptide Nanofiber Scaffold for Brain Repair and Axon Regeneration with Functional Return of Vision. Proc. Natl. Acad. Sci. 103 (13), 5054–5059. 10.1073/pnas.0600559103 16549776 PMC1405623

[B29] Ellison C. J. Phatak A. Giles D. W. Macosko C. W. Bates F. S. (2007). Melt Blown Nanofibers: Fiber Diameter Distributions and Onset of Fiber Breakup. Polymer 48 (11), 3306–3316. 10.1016/j.polymer.2007.04.005

[B30] Fan L. Liu C. Chen X. Zou Y. Zhou Z. Lin C. (2018). Directing Induced Pluripotent Stem Cell Derived Neural Stem Cell Fate with a Three-Dimensional Biomimetic Hydrogel for Spinal Cord Injury Repair. ACS Appl. Mater. Inter. 10 (21), 17742–17755. 10.1021/acsami.8b05293 29733569

[B31] Fan L. Liu C. Chen X. Zou Y. Zhou Z. Lin C. (2018). Directing Induced Pluripotent Stem Cell Derived Neural Stem Cell Fate with a Three-Dimensional Biomimetic Hydrogel for Spinal Cord Injury Repair. ACS Appl. Mater. Inter. 10 (21), 17742–17755. 10.1021/acsami.8b05293 29733569

[B32] Fehlings M. G. Wilson J. R. Harrop J. S. Kwon B. K. Tetreault L. A. Arnold P. M. (2017). Efficacy and Safety of Methylprednisolone Sodium Succinate in Acute Spinal Cord Injury: A Systematic Review. Glob. Spine J. 7 (3 Suppl. l), 116S–137S. 10.1177/2192568217706366 PMC5684849 29164020

[B33] Fennessey S. F. Farris R. J. (2004). Fabrication of Aligned and Molecularly Oriented Electrospun Polyacrylonitrile Nanofibers and the Mechanical Behavior of Their Twisted Yarns. Polymer 45 (12), 4217–4225. 10.1016/j.polymer.2004.04.001

[B34] Fong H. Liu W. D. Wang C. S. Vaia R. A. (2002). Generation of Electrospun Fibers of Nylon 6 and Nylon 6-montmorillonite Nanocomposite. Polymer 43 (3), 775–780. 10.1016/S0032-3861(01)00665-6

[B35] Fujita Y. Yamashita T. (2014). Axon Growth Inhibition by RhoA/ROCK in the central Nervous System. Front. Neurosci. 8, 338. 10.3389/fnins.2014.00338 25374504 PMC4205828

[B36] Gautier S. E. Oudega M. Fragoso M. Chapon P. Plant G. W. Bunge M. B (1998). Poly(?-hydroxyacids) for Application in the Spinal Cord: Resorbability and Biocompatibility with Adult Rat Schwann Cells and Spinal Cord. J. Biomed. Mater. Res. 42 (4), 642–654. 10.1002/(sici)1097-4636(19981215)42:4<642:aid-jbm22>3.0.co;2-k 9827690

[B37] Gelain F. Panseri S. Antonini S. Cunha C. Donega M. Lowery J. (2011). Transplantation of Nanostructured Composite Scaffolds Results in the Regeneration of Chronically Injured Spinal Cords. ACS Nano 5 (1), 227–236. 10.1021/nn102461w 21189038

[B38] Gnavi S. Fornasari B. Tonda-Turo C. Laurano R. Zanetti M. Ciardelli G. (2015). The Effect of Electrospun Gelatin Fibers Alignment on Schwann Cell and Axon Behavior and Organization in the Perspective of Artificial Nerve Design. Ijms 16 (6), 12925–12942. 10.3390/ijms160612925 26062130 PMC4490479

[B39] Grossman S. D. Wolfe B. B. Yasuda R. P. Wrathall J. R. (2000). Changes in NMDA Receptor Subunit Expression in Response to Contusive Spinal Cord Injury. J. Neurochem. 75 (1), 174–184. 10.1046/j.1471-4159.2000.0750174.x 10854260

[B40] Gwak S.-J. Yun Y. Yoon D. H. Kim K. N. Ha Y. (2016). Therapeutic Use of 3β-[N-(N′,N′-Dimethylaminoethane) Carbamoyl] Cholesterol-Modified PLGA Nanospheres as Gene Delivery Vehicles for Spinal Cord Injury. Plos One 11 (1), e0147389. 10.1371/journal.pone.0147389 26824765 PMC4732605

[B41] Habibizadeh M. Nadri S. Fattahi A. Rostamizadeh K. Mohammadi P. Andalib S. (2021). Surface Modification of Neurotrophin‐3 Loaded PCL/chitosan Nanofiber/net by Alginate Hydrogel Microlayer for Enhanced Biocompatibility in Neural Tissue Engineering. J. Biomed. Mater. Res. 109 (11), 2237–2254. 10.1002/jbm.a.37208 34132482

[B42] Hachem L. D. Ahuja C. S. Fehlings M. G. (2017). Assessment and Management of Acute Spinal Cord Injury: From point of Injury to Rehabilitation. J. Spinal Cord Med. 40 (6), 665–675. 10.1080/10790268.2017.1329076 28571527 PMC5778930

[B43] Hall A. G. Karabukayeva A. Rainey C. Kelly R. J. Patterson J. Wade J. (2021). Perspectives on Life Following a Traumatic Spinal Cord Injury. Disabil. Health J. 14 (3), 101067. 10.1016/j.dhjo.2021.101067 33722578

[B44] Hosseini M. Yousefifard M. Baikpour M. Rahimi-Movaghar V. Nasirinezhad F. Younesian S. (2016). The Efficacy of Schwann Cell Transplantation on Motor Function Recovery after Spinal Cord Injuries in Animal Models: A Systematic Review and Meta-Analysis. J. Chem. Neuroanat. 78, 102–111. 10.1016/j.jchemneu.2016.09.002 27609084

[B45] Hu Q. Sun W. Lu Y. Bomba H. N. Ye Y. Jiang T. (2016). Tumor Microenvironment-Mediated Construction and Deconstruction of Extracellular Drug-Delivery Depots. Nano Lett. 16 (2), 1118–1126. 10.1021/acs.nanolett.5b04343 26785163

[B46] Huang G. Li F. Zhao X. Ma Y. Li Y. Lin M. (2017). Functional and Biomimetic Materials for Engineering of the Three-Dimensional Cell Microenvironment. Chem. Rev. 117 (20), 12764–12850. 10.1021/acs.chemrev.7b00094 28991456 PMC6494624

[B47] Hurtado A. Cregg J. M. Wang H. B. Wendell D. F. Oudega M. Gilbert R. J. (2011). Robust CNS Regeneration after Complete Spinal Cord Transection Using Aligned Poly-L-Lactic Acid Microfibers. Biomaterials 32 (26), 6068–6079. 10.1016/j.biomaterials.2011.05.006 21636129 PMC4163047

[B48] Hurtado A. Moon L. D. F. Maquet V. Blits B. Jérôme R. Oudega M. (2006). Poly (D,L-lactic Acid) Macroporous Guidance Scaffolds Seeded with Schwann Cells Genetically Modified to Secrete a Bi-functional Neurotrophin Implanted in the Completely Transected Adult Rat Thoracic Spinal Cord. Biomaterials 27 (3), 430–442. 10.1016/j.biomaterials.2005.07.014 16102815

[B49] Hwang D. H. Kim H. M. Kang Y. M. Joo I. S. Cho C.-S. Yoon B.-W. (2011). Combination of Multifaceted Strategies to Maximize the Therapeutic Benefits of Neural Stem Cell Transplantation for Spinal Cord Repair. Cel Transpl. 20 (9), 1361–1380. 10.3727/096368910X557155 21396156

[B50] Hyysalo A. Ristola M. Joki T. Honkanen M. Vippola M. Narkilahti S. (2017). Aligned Poly(ε-Caprolactone) Nanofibers Guide the Orientation and Migration of Human Pluripotent Stem Cell-Derived Neurons, Astrocytes, and Oligodendrocyte Precursor Cells *In Vitro* . Macromol. Biosci. 17 (7), 1600517. 10.1002/mabi.201600517 28296144

[B51] Jain N. B. Ayers G. D. Peterson E. N. Harris M. B. Morse L. O’Connor K. C. (2015). Traumatic Spinal Cord Injury in the United States, 1993-2012. JAMA 313 (22), 2236–2243. 10.1001/jama.2015.6250 26057284 PMC4712685

[B52] Jazayeri S. B. Beygi S. Shokraneh F. Hagen E. M. Rahimi-Movaghar V. (2015). Incidence of Traumatic Spinal Cord Injury Worldwide: a Systematic Review. Eur. Spine J. 24 (5), 905–918. 10.1007/s00586-014-3424-6 24952008

[B53] Kakade M. V. Givens S. Gardner K. Lee K. H. Chase D. B. Rabolt J. F. (2007). Electric Field Induced Orientation of Polymer Chains in Macroscopically Aligned Electrospun Polymer Nanofibers. J. Am. Chem. Soc. 129 (10), 2777–2782. 10.1021/ja065043f 17302411

[B54] KarbalaeiMahdi A. Shahrousvand M. Javadi H. R. Ghollasi M. Norouz F. Kamali M. (2017). Neural Differentiation of Human Induced Pluripotent Stem Cells on Polycaprolactone/gelatin Bi-electrospun Nanofibers. Mater. Sci. Eng. C 78, 1195–1202. 10.1016/j.msec.2017.04.083 28575957

[B55] Keefe K. Sheikh I. Smith G. (2017). Targeting Neurotrophins to Specific Populations of Neurons: NGF, BDNF, and NT-3 and Their Relevance for Treatment of Spinal Cord Injury. Ijms 18 (3), 548. 10.3390/ijms18030548 PMC5372564 28273811

[B56] Kim H. Tator C. H. Shoichet M. S. (2011). Chitosan Implants in the Rat Spinal Cord: Biocompatibility and Biodegradation. J. Biomed. Mater. Res. 97A (4), 395–404. 10.1002/jbm.a.33070 21465644

[B57] Kim T. G. Park T. G. (2006). Biomimicking Extracellular Matrix: Cell Adhesive RGD Peptide Modified Electrospun Poly(D,L-lactic-co-glycolic Acid) Nanofiber Mesh. Tissue Eng. 12 (2), 221–233. 10.1089/ten.2006.12.221 16548681

[B58] Koppes R. A. Park S. Hood T. Jia X. Abdolrahim Poorheravi N. Achyuta A. H. (2016). Thermally Drawn Fibers as Nerve Guidance Scaffolds. Biomaterials 81, 27–35. 10.1016/j.biomaterials.2015.11.063 26717246 PMC5134911

[B59] Kriebel A. Hodde D. Kuenzel T. Engels J. Brook G. Mey J. (2017). Cell-free Artificial Implants of Electrospun Fibres in a Three-Dimensional Gelatin Matrix Support Sciatic Nerve Regeneration *In Vivo* . J. Tissue Eng. Regen. Med. 11 (12), 3289–3304. 10.1002/term.2237 28127889

[B60] Kwon B. Tetzlaff W. Grauer J. N. Beiner J. Vaccaro A. R. (2004). Pathophysiology and Pharmacologic Treatment of Acute Spinal Cord Injury*1. Spine J. 4 (4), 451–464. 10.1016/j.spinee.2003.07.007 15246307

[B61] Labroo P. Shea J. Edwards K. Ho S. Davis B. Sant H. (2017). Novel Drug Delivering Conduit for Peripheral Nerve Regeneration. J. Neural Eng. 14 (6), 066011. 10.1088/1741-2552/aa867d 28829045

[B62] Lan L. Tian F.-R. ZhuGe D.-L. ZhuGe Q.-C. Shen B.-X. Jin B.-H. (2017). Implantable Porous Gelatin Microspheres Sustained Release of bFGF and Improved its Neuroprotective Effect on Rats after Spinal Cord Injury. Plos One 12 (3), e0173814. 10.1371/journal.pone.0173814 28291798 PMC5349659

[B63] Lan L. Tian F.-R. Zhuge D.-L. Zhuge Q.-C. Shen B.-X. Jin B.-H. (2017). Implantable Porous Gelatin Microspheres Sustained Release of bFGF and Improved its Neuroprotective Effect on Rats after Spinal Cord Injury. Plos One 12 (3), e0173814. 10.1371/journal.pone.0173814 28291798 PMC5349659

[B64] Lee J. Y. Bashur C. A. Goldstein A. S. Schmidt C. E. (2009). Polypyrrole-coated Electrospun PLGA Nanofibers for Neural Tissue Applications. Biomaterials 30 (26), 4325–4335. 10.1016/j.biomaterials.2009.04.042 19501901 PMC2713816

[B65] Li G. Che M.-T. Zeng X. Qiu X.-C. Feng B. Lai B.-Q. (2018). Neurotrophin-3 Released from Implant of Tissue-Engineered Fibroin Scaffolds Inhibits Inflammation, Enhances Nerve Fiber Regeneration, and Improves Motor Function in Canine Spinal Cord Injury. J. Biomed. Mater. Res. 106 (8), 2158–2170. 10.1002/jbm.a.36414 PMC6055812 29577604

[B66] Li L. Zhou G. Wang Y. Yang G. Ding S. Zhou S. (2015). Controlled Dual Delivery of BMP-2 and Dexamethasone by Nanoparticle-Embedded Electrospun Nanofibers for the Efficient Repair of Critical-Sized Rat Calvarial Defect. Biomaterials 37, 218–229. 10.1016/j.biomaterials.2014.10.015 25453952

[B67] Li X. Dai J. (2018). Bridging the gap with Functional Collagen Scaffolds: Tuning Endogenous Neural Stem Cells for Severe Spinal Cord Injury Repair. Biomater. Sci. 6 (2), 265–271. 10.1039/c7bm00974g 29265131

[B68] Li Y. Yu Z. Men Y. Chen X. Wang B. (2018). Lamininchitosan PLGA Conduit Cotransplanted with Schwann and Neural Stem Cells to Repair the Injured Recurrent Laryngeal Nerve. Exp. Ther. Med. 16 (2), 1250–1258. 10.3892/etm.2018.6343 30116376 PMC6090254

[B69] Libro R. Bramanti P. Mazzon E. (2017). The Combined Strategy of Mesenchymal Stem Cells and Tissue-Engineered Scaffolds for Spinal Cord Injury Regeneration. Exp. Ther. Med. 14 (4), 3355–3368. 10.3892/etm.2017.4939 29042919 PMC5639409

[B70] Lim W. L. Chowdhury S. R. Ng M. H. Law J. X. (2021). Physicochemical Properties and Biocompatibility of Electrospun Polycaprolactone/Gelatin Nanofibers. Ijerph 18 (9), 4764. 10.3390/ijerph18094764 33947053 PMC8125554

[B71] Lin Y. P. Lin S. Y. Lee Y. C. Chen-Yang Y. W. (2013). High Surface Area Electrospun Prickle-like Hierarchical Anatase TiO2 Nanofibers for Dye-Sensitized Solar Cell Photoanodes. J. Mater. Chem. A. 1 (34), 9875–9884. 10.1039/c3ta10925a

[B72] Liu D. Li X. Xiao Z. Yin W. Zhao Y. Tan J. (2019). Different Functional Bio-Scaffolds Share Similar Neurological Mechanism to Promote Locomotor Recovery of Canines with Complete Spinal Cord Injury. Biomaterials 214, 119230. 10.1016/j.biomaterials.2019.119230 31174066

[B73] Liu S.-H. Liu M. Xu Z.-L. Wei Y.-M. Guo X. (2017). A Novel PES-TiO2 Hollow Fiber Hybrid Membrane Prepared via Sol-Gel Process Assisted Reverse Thermally Induced Phase Separation (RTIPS) Method. J. Membr. Sci. 528, 303–315. 10.1016/j.memsci.2017.01.028

[B74] Liu Y. Goebl J. Yin Y. (2013). Templated Synthesis of Nanostructured Materials. Chem. Soc. Rev. 42 (7), 2610–2653. 10.1039/c2cs35369e 23093173

[B75] Lv D. Zhou L. Zheng X. Hu Y. (2017). Sustained Release of Collagen VI Potentiates Sciatic Nerve Regeneration by Modulating Macrophage Phenotype. Eur. J. Neurosci. 45 (10), 1258–1267. 10.1111/ejn.13558 28263445

[B76] Mahoney M. J. Krewson C. Miller J. Saltzman W. M. (2006). Impact of Cell Type and Density on Nerve Growth Factor Distribution and Bioactivity in 3-dimensional Collagen Gel Cultures. Tissue Eng. 12 (7), 1915–1927. 10.1089/ten.2006.12.1915 16889521

[B77] Matsuyama H. Yuasa M. Kitamura Y. Teramoto M. Lloyd D. R. (2000). Structure Control of Anisotropic and Asymmetric Polypropylene Membrane Prepared by Thermally Induced Phase Separation. J. Membr. Sci. 179 (1-2), 91–100. 10.1016/s0376-7388(00)00506-8

[B78] McCullen S. D. Stevens D. R. Roberts W. A. Ojha S. S. Clarke L. I. Gorga R. E. (2007). Morphological, Electrical, and Mechanical Characterization of Electrospun Nanofiber Mats Containing Multiwalled Carbon Nanotubes. Macromolecules 40 (4), 997–1003. 10.1021/ma061735c

[B79] Meinel A. J. Germershaus O. Luhmann T. Merkle H. P. Meinel L. (2012). Electrospun Matrices for Localized Drug Delivery: Current Technologies and Selected Biomedical Applications. Eur. J. Pharmaceutics Biopharmaceutics 81 (1), 1–13. 10.1016/j.ejpb.2012.01.016 22342778

[B80] Mohtaram N. K. Ko J. King C. Sun L. Muller N. Jun M. B.-G. (2015). Electrospun Biomaterial Scaffolds with Varied Topographies for Neuronal Differentiation of Human-Induced Pluripotent Stem Cells. J. Biomed. Mater. Res. 103 (8), 2591–2601. 10.1002/jbm.a.35392 25524598

[B81] Ning G.-Z. Yu T.-Q. Feng S.-Q. Zhou X.-H. Ban D.-X. Liu Y. (2011). Epidemiology of Traumatic Spinal Cord Injury in Tianjin, China. Spinal Cord 49 (3), 386–390. 10.1038/sc.2010.130 20921958

[B82] Nomura H. Tator C. H. Shoichet M. S. (2006). Bioengineered Strategies for Spinal Cord Repair. J. Neurotrauma 23 (3-4), 496–507. 10.1089/neu.2006.23.496 16629632

[B83] Oyinbo C. A. (2011). Secondary Injury Mechanisms in Traumatic Spinal Cord Injury: a Nugget of This Multiply cascade. Acta Neurobiol. Exp. (Wars) 71 (2), 281–299. 21731081 10.55782/ane-2011-1848

[B84] Pan S. Qi Z. Li Q. Ma Y. Fu C. Zheng S. (2019). Graphene Oxide-PLGA Hybrid Nanofibres for the Local Delivery of IGF-1 and BDNF in Spinal Cord Repair. Artif. Cell Nanomedicine, Biotechnol. 47 (1), 650–663. 10.1080/21691401.2019.1575843 30829545

[B85] Pan S. Zhao Y. Qiao X. Qi Z. Fu C. Kong W. (2019). PLGA Porous Scaffolds by Polydopamine-Assisted Immobilization of NGF for Spinal Cord Injury Repair. Mater. Res. Express 6 (4), 045024. 10.1088/2053-1591/aafa8a

[B86] Pang M. Shu T. Chen R. Q. Liu C. He L. Yang Y. (2016). Neural Precursor Cells Generated from Induced Pluripotent Stem Cells with Gelatin Sponge-Electrospun PLGA/PEG Nanofibers for Spinal Cord Injury Repair. Int. J. Clin. Exp. Med. 9 (9), 17985–17994.

[B87] Park J. H. Kim B. S. Yoo Y. C. Khil M. S. Kim H. Y. (2008). Enhanced Mechanical Properties of Multilayer Nano-Coated Electrospun Nylon 6 Fibers via a Layer-By-Layer Self-Assembly. J. Appl. Polym. Sci. 107 (4), 2211–2216. 10.1002/app.27322

[B88] Patel B. B. Sharifi F. Stroud D. P. Montazami R. Hashemi N. N. Sakaguchi D. S. (2019). 3D Microfibrous Scaffolds Selectively Promotes Proliferation and Glial Differentiation of Adult Neural Stem Cells: A Platform to Tune Cellular Behavior in Neural Tissue Engineering. Macromol. Biosci. 19 (2), 1800236. 10.1002/mabi.201800236 30480879

[B89] Petcu E. B. Midha R. McColl E. Popa-Wagner A. Chirila T. V. Dalton P. D. (2018). 3D Printing Strategies for Peripheral Nerve Regeneration. Biofabrication 10 (3), 032001. 10.1088/1758-5090/aaaf50 29570458

[B90] Pivovarova N. B. Andrews S. B. (2010). Calcium-dependent Mitochondrial Function and Dysfunction in Neurons. FEBS J. 277 (18), 3622–3636. 10.1111/j.1742-4658.2010.07754.x 20659161 PMC3489481

[B91] Rahimi-Movaghar V. Sayyah M. K. Akbari H. Khorramirouz R. Rasouli M. R. Moradi-Lakeh M. (2013). Epidemiology of Traumatic Spinal Cord Injury in Developing Countries: a Systematic Review. Neuroepidemiology 41 (2), 65–85. 10.1159/000350710 23774577

[B92] Rao J.-S. Zhao C. Zhang A. Duan H. Hao P. Wei R.-H. (2018). NT3-chitosan Enables De Novo Regeneration and Functional Recovery in Monkeys after Spinal Cord Injury. Proc. Natl. Acad. Sci. USA 115 (24), E5595–E5604. 10.1073/pnas.1804735115 29844162 PMC6004491

[B93] Reis K. P. Sperling L. E. Teixeira C. Paim Á. Alcântara B. Vizcay-Barrena G. (2018). Application of PLGA/FGF-2 Coaxial Microfibers in Spinal Cord Tissue Engineering: an *In Vitro* and *In Vivo* Investigation. Regenerative Med. 13 (7), 785–801. 10.2217/rme-2018-0060 30289057

[B94] Reneker D. H. Kataphinan W. Theron A. Zussman E. Yarin A. L. (2002). Nanofiber Garlands of Polycaprolactone by Electrospinning. Polymer 43 (25), 6785–6794. 10.1016/S0032-3861(02)00595-5

[B95] Reneker D. H. Yarin A. L. Fong H. Koombhongse S. (2000). Bending Instability of Electrically Charged Liquid Jets of Polymer Solutions in Electrospinning. J. Appl. Phys. 87 (9), 4531–4547. 10.1063/1.373532

[B96] Safa B. Buncke G. (2016). Autograft Substitutes. Hand Clin. 32 (2), 127–140. 10.1016/j.hcl.2015.12.012 27094886

[B97] Schiffman J. D. Schauer C. L. (2008). A Review: Electrospinning of Biopolymer Nanofibers and Their Applications. Polym. Rev. 48 (2), 317–352. 10.1080/15583720802022182

[B98] Schmidt C. E. Leach J. B. (2003). Neural Tissue Engineering: Strategies for Repair and Regeneration. Annu. Rev. Biomed. Eng. 5, 293–347. 10.1146/annurev.bioeng.5.011303.120731 14527315

[B99] Shahriari D. Koffler J. Y. Tuszynski M. H. Campana W. M. Sakamoto J. S. (2017). Hierarchically Ordered Porous and High-Volume Polycaprolactone Microchannel Scaffolds Enhanced Axon Growth in Transected Spinal Cords. Tissue Eng. Part A 23 (9-10), 415–425. 10.1089/ten.tea.2016.0378 28107810 PMC5444512

[B100] Shi D. He T. Tang W. Li H. Wang C. Zheng M. (2019). Local Application of MDL28170-Loaded PCL Film Improves Functional Recovery by Preserving Survival of Motor Neurons after Traumatic Spinal Cord Injury. Neurosci. Lett. 694, 161–167. 10.1016/j.neulet.2018.12.006 30528875

[B101] Shu B. Sun X. Liu R. Jiang F. Yu H. Xu N. (2019). Restoring Electrical Connection Using a Conductive Biomaterial Provides a New Therapeutic Strategy for Rats with Spinal Cord Injury. Neurosci. Lett. 692, 33–40. 10.1016/j.neulet.2018.10.031 30367954

[B102] Silantyeva E. A. Nasir W. Carpenter J. Manahan O. Becker M. L. Willits R. K. (2018). Accelerated Neural Differentiation of Mouse Embryonic Stem Cells on Aligned GYIGSR-Functionalized Nanofibers. Acta Biomater. 75, 129–139. 10.1016/j.actbio.2018.05.052 29879551 PMC6774047

[B103] Sugai K. Nishimura S. Kato-Negishi M. Onoe H. Iwanaga S. Toyama Y. (2015). Neural Stem/progenitor Cell-Laden Microfibers Promote Transplant Survival in a Mouse Transected Spinal Cord Injury Model. J. Neurosci. Res. 93 (12), 1826–1838. 10.1002/jnr.23636 26301451

[B104] Sun F. Shi T. Zhou T. Dong D. Xie J. Wang R. (2017). 3D Poly(Lactic-Co-Glycolic Acid) Scaffolds for Treating Spinal Cord Injury. J Biomed. Nanotechnol 13 (3), 290–302. 10.1166/jbn.2017.2348 29381284

[B105] Sun G. D. Shao J. L. Deng D. J. Zhou Z. G. Zhou X. B. Lin Y. X. (2017). A Chitosan Scaffold Infused with Neurotrophin-3 and Human Umbilical Cord Mesenchymal Stem Cells Suppresses Inflammation and Promotes Functional Recovery after Spinal Cord Injury in Mice. Int. J. Clin. Exp. Med. 10 (8), 11672–11679.

[B106] Sun Y. Yang C. Zhu X. Wang J. J. Liu X. Y. Yang X. P. (2019). 3D Printing Collagen/chitosan Scaffold Ameliorated Axon Regeneration and Neurological Recovery after Spinal Cord Injury. J. Biomed. Mater. Res. 107 (9), 1898–1908. 10.1002/jbm.a.36675 30903675

[B107] Tao J. Zhang J. Du T. Xu X. Deng X. Chen S. (2019). Rapid 3D Printing of Functional Nanoparticle-Enhanced Conduits for Effective Nerve Repair. Acta Biomater. 90, 49–59. 10.1016/j.actbio.2019.03.047 30930306

[B108] Tator C. H. (1995). Update on the Pathophysiology and Pathology of Acute Spinal Cord Injury. Brain Pathol. 5 (4), 407–413. 10.1111/j.1750-3639.1995.tb00619.x 8974623

[B109] Terraf P. Kouhsari S. M. Ai J. Babaloo H. (2017). Tissue-Engineered Regeneration of Hemisected Spinal Cord Using Human Endometrial Stem Cells, Poly ε-Caprolactone Scaffolds, and Crocin as a Neuroprotective Agent. Mol. Neurobiol. 54 (7), 5657–5667. 10.1007/s12035-016-0089-7 27624387

[B110] Tian L. Prabhakaran M. P. Ramakrishna S. (2015). Strategies for Regeneration of Components of Nervous System: Scaffolds, Cells and Biomolecules. Regenerative Biomater. 2 (1), 31–45. 10.1093/rb/rbu017 PMC4669026 26813399

[B111] Tran A. P. Warren P. M. Silver J. (2018). The Biology of Regeneration Failure and Success after Spinal Cord Injury. Physiol. Rev. 98 (2), 881–917. 10.1152/physrev.00017.2017 29513146 PMC5966716

[B112] Ursavas S. Darici H. Karaoz E. (2021). Olfactory Ensheathing Cells: Unique Glial Cells Promising for Treatments of Spinal Cord Injury. J. Neurosci. Res. 99 (6), 1579–1597. 10.1002/jnr.24817 33605466

[B113] Uygun B. E. Yarmush M. L. Uygun K. (2012). Application of Whole-Organ Tissue Engineering in Hepatology. Nat. Rev. Gastroenterol. Hepatol. 9 (12), 738–744. 10.1038/nrgastro.2012.140 22890112 PMC3732057

[B114] Valmikinathan C. M. Defroda S. Yu X. (2009). Polycaprolactone and Bovine Serum Albumin Based Nanofibers for Controlled Release of Nerve Growth Factor. Biomacromolecules 10 (5), 1084–1089. 10.1021/bm8012499 19323510

[B115] von Leden R. E. Yauger Y. J. Khayrullina G. Byrnes K. R. (2017). Central Nervous System Injury and Nicotinamide Adenine Dinucleotide Phosphate Oxidase: Oxidative Stress and Therapeutic Targets. J. Neurotrauma 34 (4), 755–764. 10.1089/neu.2016.4486 27267366 PMC5335782

[B116] Wang C. Sun C. Hu Z. Huo X. Yang Y. Liu X. (2017). Improved Neural Regeneration with Olfactory Ensheathing Cell Inoculated PLGA Scaffolds in Spinal Cord Injury Adult Rats. Neurosignals 25 (1), 1–14. 10.1159/000471828 28359049

[B117] Wang J. Wang J. Lu P. Cai Y. Wang Y. Hong L. (2015). Local Delivery of FTY720 in PCL Membrane Improves SCI Functional Recovery by Reducing Reactive Astrogliosis. Biomaterials 62, 76–87. 10.1016/j.biomaterials.2015.04.060 26036174

[B118] Wang N. Xiao Z. Zhao Y. Wang B. Li X. Li J. (2018). Collagen Scaffold Combined with Human Umbilical Cord‐derived Mesenchymal Stem Cells Promote Functional Recovery after Scar Resection in Rats with Chronic Spinal Cord Injury. J. Tissue Eng. Regen. Med. 12 (2), e1154–e1163. 10.1002/term.2450 28482124

[B119] Wang W. Itoh S. Konno K. Kikkawa T. Ichinose S. Sakai K. (2009). Effects of Schwann Cell Alignment along the Oriented Electrospun Chitosan Nanofibers on Nerve Regeneration. J. Biomed. Mater. Res. 91A (4), 994–1005. 10.1002/jbm.a.32329 19097155

[B120] Wen Y. Yu S. Wu Y. Ju R. Wang H. Liu Y. (2016). Spinal Cord Injury Repair by Implantation of Structured Hyaluronic Acid Scaffold with PLGA Microspheres in the Rat. Cell Tissue Res 364 (1), 17–28. 10.1007/s00441-015-2298-1 26463048

[B121] Wilson J. R. Singh A. Craven C. Verrier M. C. Drew B. Ahn H. (2012). Early versus Late Surgery for Traumatic Spinal Cord Injury: the Results of a Prospective Canadian Cohort Study. Spinal Cord 50 (11), 840–843. 10.1038/sc.2012.59 22565550

[B122] Wilson J. R. Tetreault L. A. Kwon B. K. Arnold P. M. Mroz T. E. Shaffrey C. (2017). Timing of Decompression in Patients with Acute Spinal Cord Injury: A Systematic Review. Glob. Spine J. 7 (3 Suppl. l), 95S–115S. 10.1177/2192568217701716 PMC5684838 29164038

[B123] Wissink M. J. B. Beernink R. Pieper J. S. Poot A. A. Engbers G. H. M. Beugeling T. (2001). Immobilization of Heparin to EDC/NHS-crosslinked Collagen. Characterization and *In Vitro* Evaluation. Biomaterials 22 (2), 151–163. 10.1016/s0142-9612(00)00164-2 11101159

[B124] Wittmer C. R. Claudepierre T. Reber M. Wiedemann P. Garlick J. A. Kaplan D. (2011). Multifunctionalized Electrospun Silk Fibers Promote Axon Regeneration in the Central Nervous System. Adv. Funct. Mater. 21 (22), 4232–4242. 10.1002/adfm.201100755 PMC3404853 22844266

[B125] Wu H.-g. Yao Z.-a. Chen F.-j. Cui H.-l. Guo T. N. Wu H. G. (2018). Efficacy of Chitosan and Sodium Alginate Scaffolds for Repair of Spinal Cord Injury in Rats. Neural Regen. Res. 13 (3), 502–509. 10.4103/1673-5374.228756 29623937 PMC5900515

[B126] Xie J. MacEwan M. R. Schwartz A. G. Xia Y. (2010). Electrospun Nanofibers for Neural Tissue Engineering. Nanoscale 2 (1), 35–44. 10.1039/b9nr00243j 20648362

[B127] Xu X. Zhuang X. Chen X. Wang X. Yang L. Jing X. (2006). Preparation of Core-Sheath Composite Nanofibers by Emulsion Electrospinning. Macromol. Rapid Commun. 27 (19), 1637–1642. 10.1002/marc.200600384

[B128] Xue W. Fan C. Chen B. Zhao Y. Xiao Z. Dai J. (2021). Direct Neuronal Differentiation of Neural Stem Cells for Spinal Cord Injury Repair. Stem Cells 39 (8), 1025–1032. 10.1002/stem.3366 33657255

[B129] Yan L. Ji J. Xie D. Li W. Zhang G. (2008). Surfactant-free Synthesis of Amphiphilic Copolymer of Poly(styrene-Co-Acrylamide) in Aqueous Emulsion with the Assistance of Ultrasound. Polym. Adv. Technol. 19 (3), 221–228. 10.1002/pat.1000

[B130] Yang F. Murugan R. Ramakrishna S. Wang X. Ma Y.-X. Wang S. (2004). Fabrication of Nano-Structured Porous PLLA Scaffold Intended for Nerve Tissue Engineering. Biomaterials 25 (10), 1891–1900. 10.1016/j.biomaterials.2003.08.062 14738853

[B131] Yang F. Murugan R. Wang S. Ramakrishna S. (2005). Electrospinning of Nano/micro Scale poly(L-Lactic Acid) Aligned Fibers and Their Potential in Neural Tissue Engineering. Biomaterials 26 (15), 2603–2610. 10.1016/j.biomaterials.2004.06.051 15585263

[B132] Yang Z. Zhang A. Duan H. Zhang S. Hao P. Ye K. (2015). NT3-chitosan Elicits Robust Endogenous Neurogenesis to Enable Functional Recovery after Spinal Cord Injury. Proc. Natl. Acad. Sci. USA 112 (43), 13354–13359. 10.1073/pnas.1510194112 26460015 PMC4629318

[B133] Yang Z. Zhang A. Duan H. Zhang S. Hao P. Ye K. (2015). NT3-chitosan Elicits Robust Endogenous Neurogenesis to Enable Functional Recovery after Spinal Cord Injury. Proc. Natl. Acad. Sci. USA 112 (43), 13354–13359. 10.1073/pnas.1510194112 26460015 PMC4629318

[B134] Yin W. Li X. Zhao Y. Tan J. Wu S. Cao Y. (2018). Taxol-modified Collagen Scaffold Implantation Promotes Functional Recovery after Long-Distance Spinal Cord Complete Transection in Canines. Biomater. Sci. 6 (5), 1099–1108. 10.1039/c8bm00125a 29528079

[B135] Zhang D. Wang Q. Wang S. Huang Y. Tian N. Wu Y. (2019). Astragoloside IV Loaded Polycaprolactone Membrane Repairs Blood Spinal Cord Barrier and Recovers Spinal Cord Function in Traumatic Spinal Cord Injury. J Biomed. Nanotechnol 15 (4), 799–812. 10.1166/jbn.2019.2715 30841972

[B136] Zhang J. Cheng T. Chen Y. Gao F. Guan F. Yao M. (2020). A Chitosan-Based Thermosensitive Scaffold Loaded with Bone Marrow-Derived Mesenchymal Stem Cells Promotes Motor Function Recovery in Spinal Cord Injured Mice. Biomed. Mater. 15, 035020. 10.1088/1748-605X/ab785f 32079004

[B137] Zhang S. Wang X.-J. Li W.-S. Xu X.-L. Hu J.-B. Kang X.-Q. (2018). Polycaprolactone/polysialic Acid Hybrid, Multifunctional Nanofiber Scaffolds for Treatment of Spinal Cord Injury. Acta Biomater. 77, 15–27. 10.1016/j.actbio.2018.06.038 30126591

[B138] Zuidema J. M. Hyzinski-García M. C. Van Vlasselaer K. Zaccor N. W. Plopper G. E. Mongin A. A. (2014). Enhanced GLT-1 Mediated Glutamate Uptake and Migration of Primary Astrocytes Directed by Fibronectin-Coated Electrospun Poly-L-Lactic Acid Fibers. Biomaterials 35 (5), 1439–1449. 10.1016/j.biomaterials.2013.10.079 24246642 PMC4183153

